# Multiphase CT-based prediction of Child-Pugh classification: a machine learning approach

**DOI:** 10.1186/s41747-020-00148-3

**Published:** 2020-04-06

**Authors:** Johannes Thüring, Oliver Rippel, Christoph Haarburger, Dorit Merhof, Philipp Schad, Philipp Bruners, Christiane K. Kuhl, Daniel Truhn

**Affiliations:** 1 Department of Diagnostic and Interventional Radiology, RWTH Aachen University Hospital, Pauwelsstraße 30, 52072 Aachen, Germany; 2 Institute of Imaging and Computer Vision, RWTH Aachen University, Aachen, Germany

**Keywords:** Artificial intelligence, Liver cirrhosis, Machine learning, Neural networks (computer), Tomography (x-ray computed)

## Abstract

**Background:**

To evaluate whether machine learning algorithms allow the prediction of Child-Pugh classification on clinical multiphase computed tomography (CT).

**Methods:**

A total of 259 patients who underwent diagnostic abdominal CT (unenhanced, contrast-enhanced arterial, and venous phases) were included in this retrospective study. Child-Pugh scores were determined based on laboratory and clinical parameters. Linear regression (LR), Random Forest (RF), and convolutional neural network (CNN) algorithms were used to predict the Child-Pugh class. Their performances were compared to the prediction of experienced radiologists (ERs). Spearman correlation coefficients and accuracy were assessed for all predictive models. Additionally, a binary classification in low disease severity (Child-Pugh class A) and advanced disease severity (Child-Pugh class ≥ B) was performed.

**Results:**

Eleven imaging features exhibited a significant correlation when adjusted for multiple comparisons with Child-Pugh class. Significant correlations between predicted and measured Child-Pugh classes were observed (ρ_LA_ = 0.35, ρ_RF_ = 0.32, ρ_CNN_ = 0.51, ρ_ERs_ = 0.60; *p* < 0.001). Significantly better accuracies for the prediction of Child-Pugh classes *versus* no-information rate were found for CNN and ERs (*p* ≤ 0.034), not for LR and RF (*p* ≥ 0.384). For binary severity classification, the area under the curve at receiver operating characteristic analysis was significantly lower (*p* ≤ 0.042) for LR (0.71) and RF (0.69) than for CNN (0.80) and ERs (0.76), without significant differences between CNN and ERs (*p* = 0.144).

**Conclusions:**

The performance of a CNN in assessing Child-Pugh class based on multiphase abdominal CT images is comparable to that of ERs.

## Key points

- Established machine learning algorithms can predict the Child-Pugh class of a liver based on a clinical multiphase computed tomography.
- The predictive performance of a convolutional neural network in assessing liver parenchyma has the potential to be comparable to that of experienced radiologists.
- Machine learning algorithms, in particular convolutional neural networks, may constitute an adjunct quantitative and objective tool to assess the functional liver status based on imaging information.

## Background

Computer tomography (CT) is routinely used in the diagnosis and clinical management of patients with chronic liver disease [1, 2] and it is recognised as a sensitive diagnostic tool for evaluating morphological changes of liver parenchyma [2–4]. CT has been shown to be suitable for *in vivo* characterisation of liver cirrhosis and functionality [5, 6]. Common imaging biomarkers for the severity of liver cirrhosis are shrinkage of total liver volume, irregularity of organ boundaries, and heterogeneity of liver parenchyma; however, most of these imaging biomarkers remain unspecific [7].

To widen the value of image-based diagnosis, recent studies investigated machine learning algorithms and their potential clinical application, in particular the value of predicting biological or molecular characteristics through image-specific features [8–11]. Building on this, artificial neuronal networks have been employed to use implicit image information that might not be encompassed in dedicated human-made radiomic feature sets [10, 12].

However, accurate assessment of liver cirrhosis seems to be challenging against the background of the inherent disease heterogeneity. Hence, invasive biopsy of hepatic parenchyma is still the standard of care [13]. In an effort to overcome this potential injuring of the liver and the associated time and material consuming processes, noninvasive laboratory tests have gained importance [14]. A solid body of scientific literature still indicates serum bilirubin, albumin, or prothrombin time as the most validated and clinically used laboratory parameters regarding liver cirrhosis and changes in liver metabolism [15–17]. Moreover, in adjunction with clinical assessment, they continue to form the basis for the most widely used clinical scores for liver cirrhosis, that is, the Child-Pugh classification and the model of end-stage liver disease (MELD) [18].

Abdominal CT scans are routinely used in clinical practice and are often available for patients at risk for liver cirrhosis. However, even though a quantifiable image-based measure of liver cirrhosis beyond radiological assessment would provide a diagnostic and potentially even therapy-guiding value, it is not yet used. Moreover, even experienced radiologists (ERs) could miss subtle changes in liver parenchyma, while objective algorithms could improve the consistency of grading the liver parenchyma towards beginning cirrhosis.

In this study, we try to address this need by training and testing machine learning algorithms on routine abdominal CT scans to detect and possibly monitor patients at risk of developing liver cirrhosis noninvasively. CT-based parameters were correlated with established clinical and laboratory features from a single-institutional cohort of 259 patients. Diagnostic liver CTs of these patients were analysed by means of radiomic analysis using linear regression (LR) and random forest (RF) methods. Analysis via convolutional neural networks (CNN) was used as an additional comparison. Child-Pugh class was evaluated for each patient as an established and validated surrogate for the severity of liver cirrhosis [19, 20].

Therefore, the overarching objectives of this study were (a) to identify univariate associations between radiomic image features and Child-Pugh class in an explorative analysis; (b) to create predictive machine learning models evaluating imaging appearance for the prediction of the underlying liver cirrhosis; and (c) to compare these results to the prediction of ERs.

## Methods

### Patient population

Retrospective evaluation of imaging data was approved by the local ethics committee and informed consent was waived. The study was conducted in accordance with contemporary data protection laws. CT was performed as a part of the clinical routine of patients with abdominal diseases. A radiologist with 5 years of abdominal imaging experience (J.T.) screened the local picture archive and communication system for patients who underwent a multiphase liver CT between January 2010 and December 2016, resulting in a total of 906 patients. Exclusion criteria were as follows: (a) patients with incomplete laboratory examination records of prothrombin time, creatinine, bilirubin, and albumin within the hospitalisation period (*n* = 451); (b) presence of focal liver parenchyma changes (neoplasia *n* = 66; abscesses *n* = 41); (c) history of liver surgery or liver interventions (*n* = 89). After exclusion of these patients, a total of 259 patients served as the final cohort for this study. The CT indications for the final cohort was: staging of malignancies (*n* = 189; including 123 examinations due to hepatic cellular cancer suspicions liver lesions); infection (*n* = 47), investigation of abdominal vessels (*n* = 14), and abdominal trauma (*n* = 9).

### Child-Pugh classification

The Child-Pugh classification includes three continuous variables (prothrombin time, bilirubin, and albumin) and two discrete variables (ascites and encephalopathy). The cut-off values for all parameters were defined according to Forman et al. [18] (Table 1). Encephalopathy score was determined by transferring the daily medical bedside record into a cognitive status according to the West-Haven criteria [21, 22]. Ascites score was evaluated by measuring the perihepatic ascites expansion in the transverse plane at the portal vein bifurcation [18], measured by one radiologist with 6 years of experience in abdominal imaging (J.T.). Child-Pugh score was calculated based on the paper by Pugh et al. [23]. Patients with a score of 5 or 6 were assigned to class A, patients with scores 7–9 were assigned to class B, and patients with scores 10–15 were assigned to class C.

**Table 1:** Child-Pugh classification

	1 point	2 points	3 points
Bilirubin (mg/dL)	< 2	2–3	> 3
Albumin (g/dL)	> 3.5	2.8–3.5	< 2.8
PT prolongation (s)	1–3	4–6	> 6
Ascites (cm)	None	≤ 1	> 1
Encephalopathy	None	Mild (grades 1–2)	Severe (grades 3–4)

Child-Pugh classes: A, 5–6 points; B, 7–9 points; C, 10–15 points. *PT* Prothrombin time

### CT protocol and image postprocessing

Image acquisition parameters are summarised in Table 2. In brief, CT was performed using helical CT scanners (Somatom Definition Flash or Somatom Definiton AS, Siemens Medical Systems, Erlangen, Germany). The scans were acquired along craniocaudal direction by using a detector configuration of 128 or 40 × 0.6 mm, a tube voltage of 120 kVp, automated tube current modulation to a quality reference of 240 mAs, and online dose modulation [24] in all phases. Pitch was set to 1.0 and imaging of each phase was performed during a single breath-hold helical acquisition of roughly 10 s (according to the size of the abdomen). For all imaging, the gantry rotation speed was 0.5 s. Contrast-enhanced images were acquired following body weight–adapted application of iodinated contrast material (1.5 mL/kg of body weight; Iopromide 370 mg/mL, Ultravist, Bayer, Leverkusen, Germany) administered at a rate of 3 mL/s by a power injector. Subsequently, the unenhanced as well as hepatic arterial and portal-venous contrast phases were acquired. Computer-assisted bolus-tracking software was used to determine the optimal scan delay for each patient. The acquisition of the arterial phase started 6 s after the automatic detection of peak aortic enhancement at the level of the coeliac trunk with a threshold of 140 HU; portal venous phase was scanned 55 seconds after the start of the contrast injection. Image reconstruction was performed with axial 1-mm-thickness images, with an increment of 0.7 mm, and a B30f convolutional kernel for all phases as applied. Data were pseudo-anonymised and stored on a local hard drive.

**Table 2:** Computed tomography scan parameters

CT systems	Somatom Definiton Flash or Somatom Definiton AS
Detector configuration	128/40 × 0.6 mm
Tube voltage (kVp)	120
Exposure quality reference (mAs)	240
Pitch	1.0
Gantry rotation speed (s)	0.5
Contrast media	Iopromide 370
Application rate (mL/s)	3
Acquired phases	Unenhanced, arterial, and portal-venous
Slice thickness (mm)	1
Convolutional kernel	B30f

**Fig. 1:**
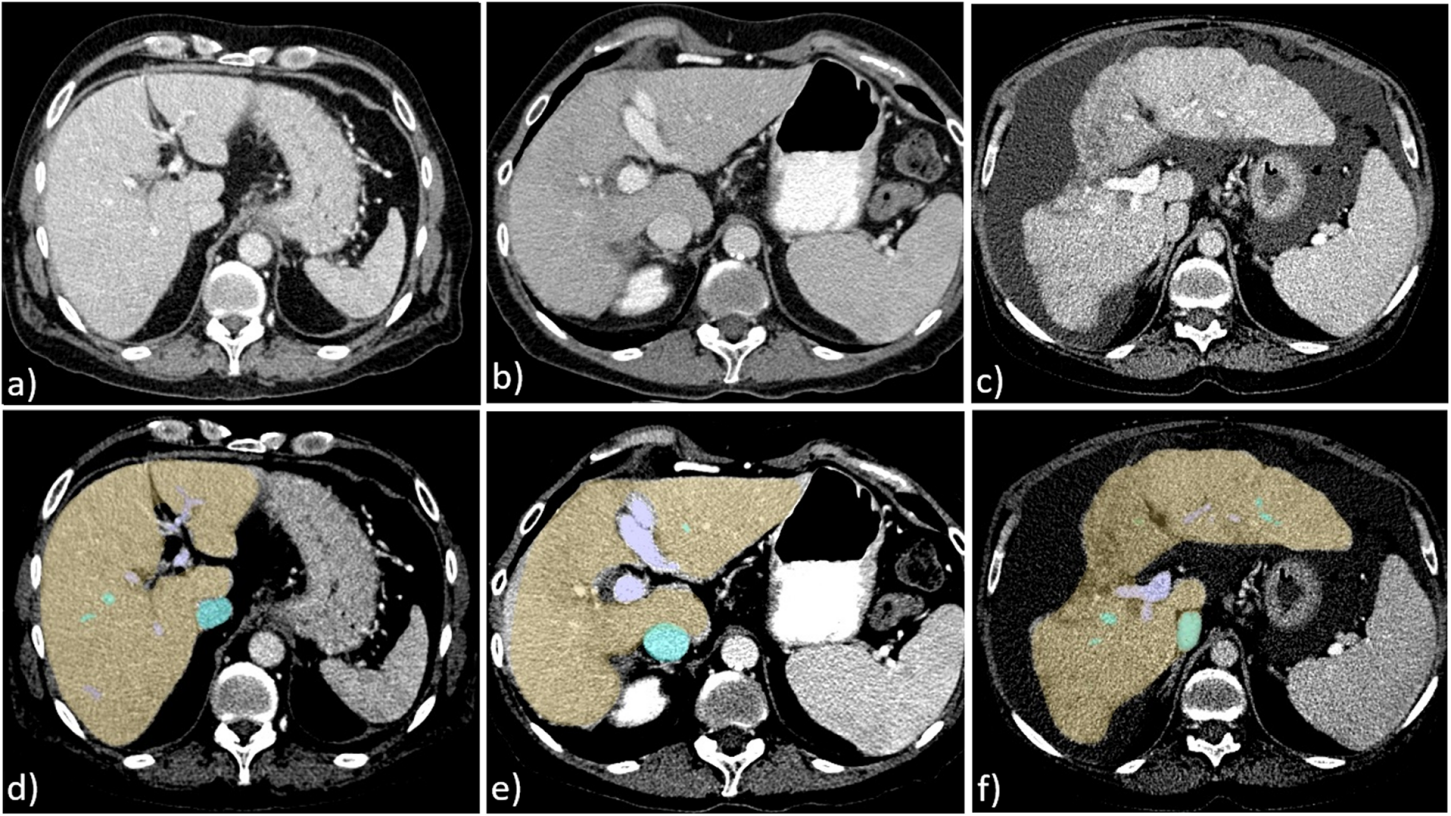
Multiphase computed tomography of three patients. **a**–**c** Transversal reconstruction of three patients in portal-venous phase. **d**–**f** Pre-processing with semiautomatic liver and vein delineation. **a**, **d** Patient with Child-Pugh class A: no changes in liver size or liver parenchyma were observed; all models rated the liver as Child-Pugh class A. **b**, **e** Patient with Child-Pugh class B: slight changes in liver configuration as well as heterogeneity of liver parenchyma were observed; only the convolutional network and the expert radiologists’ prediction rated the liver correctly as Child-Pugh class B, whereas the linear regression and the random forest rated it as a Child-Pugh class C. **c**, **f** Patient with Child-Pugh class C: overall appearance of the liver exhibits characteristic changes (liver configuration, size, and parenchyma texture); all models rated the liver as Child-Pugh class C.

All three phases were spatially co-registered to each other using a rigid transformation. The quality of registration was checked visually by one of the authors (J.T.). For liver segmentation, the venous phase was transferred to a separate workstation and analysed semiautomatically by liver volume software (Philips Intellispace Portal, Version 5.1, Philips Medical Systems, Best, the Netherlands) which encompasses automatic segmentation algorithms for liver vessels based on a deformable mesh [25]. All segmentations were checked visually and corrected manually if needed. Preprocessing was completed by a transformation of the colour-coded delineation maps into a binary evaluation mask for statistical analysis (Fig. 1).

### Radiomic feature extraction

Radiomic features comprised statistical-, shape-, and texture-based features (grey-level co-occurrence matrix, grey-level size zone matrix, grey-level run-length matrix). Features were extracted from the full liver volume using the pyradiomics framework [26]. A detailed description of all features can be found at https://pyradiomics.readthedocs.io/en/latest/features.html. In total, 271 features were extracted from the three contrast phases for each patient.

### Statistical analysis and image rating

One of the authors with more than 2 years of experience in computational biology (O.R.) performed statistical analysis. Univariate associations were evaluated between each radiomic feature (*n* = 271) and Child-Pugh class. The *p* values generated for each feature were corrected for multiple comparisons by means of family wise error rate adjustment by using the Bonferroni procedure [27].

Regarding multivariate classification, three machine learning approaches were evaluated to predict the Child-Pugh class.

#### Linear regression

Feature selection was performed in a first step by means of recursive elimination of imaging features rank based on the variance inflation factor; thresholds of 3.3, 5.0, and 10 were tested [28]. The remaining features were used for further analysis (*n* = 29) and are given in the supplemental material.

#### Random forest

Regressors were trained on the whole set of imaging features. Instead of selecting features prior to training, implicit feature selection is thus performed.

### Convolutional neural network

Instead of applying feature extraction, selection, and model training, a CNN pretrained on publicly available natural images (ImageNet; http://www.image-net.org/) was used in a transfer-learning approach to automatically predict Child-Pugh class based on two-dimensional axial slices containing the liver. To avoid slices which contain only miniscule amounts of liver tissue, we excluded the furthest 20% of slices in cranial and in caudal direction of the liver. Slice-level scores were subsequently aggregated for each patient separately by means of averaging. The detailed network architecture and training setup has been described previously [29]; in short, we use a ResNet 18 architecture as initially described [30]. Instead of using red-green-blue images, we stack the corresponding native, venous, and arterial phases of a single slice along the channel dimension, yielding a pseudo-red-green-blue image and allowing for ImageNet pretraining. This pretraining on ImageNet data was applied to the feature extraction part of the classifier and training of the classification problem at hand started with such found weights as initialisation. As a side note, we also trained the network from scratch, but found the results to be far inferior to the pretrained network architectures.

Model training was performed in a multiclass setting. The performance of each machine learning model was assessed based on a 10-fold cross-validation procedure with splits into 10 % testing-set, 27 % validation-set, and 63 % training-set. Splits were stratified such that a patient only ever belonged to one of the 3 sets.

Class imbalances were mitigated during training by subsampling in which the majority class is downsampled as previously described [31]. Each machine learning algorithm rated the imaging data with a continuous rating score (RS) ranging from 0 (corresponding to Child-Pugh class A) to 2 (corresponding to Child-Pugh class C). We chose to perform regression on the underlying Child-Pugh score first, which was then followed by classification as this approach—in contrast to a pure classification without preceding regression—accounts for the similarity between neighbouring classes. Subsequent classifications were generated by using equidistant cutoffs (RS 0.00–0.66, Child-Pugh class A; RS 0.67–1.33: Child-Pugh class B; RS = 1.34–2.00, Child-Pugh class C) rounding to the nearest class for multiclass approaches.

For the human-reader-based rating of Child-Pugh class, three experienced radiologists rated the appearance of the liver; all radiologist were blinded to the Child-Pugh score of each patient. Towards the experience of the three radiologists, each passed a standardised curricular training in a comprehensive cancer centre. One radiologist (D.T.) had more than 7 years of experience in liver imaging focused on abdominal magnetic resonance imaging, the second radiologist (J.T.) and the third radiologist (P.S.) had more than 5 years of experience in abdominal radiology and attended the oncological liver imaging circle to complete a specialised fellowship in interventional oncology. In case of a disagreement, a consensus reading with all radiologists was carried out. Interobserver agreement between the three blinded radiologists was evaluated by using Fleiss' kappa (*κ*), with results categorised according to Landis and Koch [32]. Due to the ordinal scale of the Child-Pugh class, a rank coefficient by means of Spearman ρ was used to quantify agreement between the machine learning algorithms. Moreover, the accuracy was determined for each model. The measured accuracies were tested against the no-information rate, a classifier that assigns the most prevalent class to all samples.

Finally, a binary classification of low disease severity (Child-Pugh class A) and advanced disease severity (Child-Pugh class B or C) was evaluated by means of accuracy, sensitivity and specificity. Receiver operating characteristic (ROC) analysis was performed with evaluation of the area under the curve (AUC). Testing for significance between AUCs was done by utilising bootstrapping and performing a 20,000-fold resampling. *p-*values ≤ 0.05 were regarded as statistically significant.

## Results

Epidemiologic, laboratory, and clinical characteristics are shown in Table 3. In brief, 81 female and 178 male patients with a median age of 63 years (interquartile range, 57–69 years) were included. The most frequent Child-Pugh class was B (*n* = 120; 46%), followed by A (*n* = 76; 29%) and C (*n* = 63; 24%). Elevated subscores (≥ 2) for the laboratory and clinical parameters were reported in 53% for prothrombin time, in 56 % for bilirubin, in 53 % for albumin, in 52% for ascites, and in 42% for encephalopathy.

**Table 3:** Demographic, clinical, and laboratory characteristics of 259 patients

Sex	*N*
Men	178
Women	81
Age (years)	
Median (IQR)	63 (57–69)
PT score	
1	123
2	118
3	18
Bilirubin score	
1	112
2	97
3	50
Albumin score	
1	123
2	95
3	41
Ascites score	
1	124
2	51
3	84
Encephalopathy	
1	175
2	61
3	23
Child-Pugh Class	
A	76
B	120
C	63

*PT* Prothrombin time

In total, 11 significant radiomic imaging features were found to correlate significantly with the Child-Pugh class at univariate analysis. Ten out of these were texture features and only one was shape-based (maximum two-dimensional diameter of the liver). A list of all 11 radiomic features is given in the online supplement together with their respective *p* values (supplemental Table S1).

For the choice of the variance inflation factor for linear regression, we found that a value of 5.0 performed best and used it in the following. With this threshold, 29 features were identified. Among them, 27 were texture features and only 2 were shape features (maximum extension of the liver in the ventral and lateral direction). The majority of the identified texture features originated from the arterial phase (*n* = 12), followed by the native phase (*n* = 8) and the venous phase (*n* = 7).

**Fig. 2:**
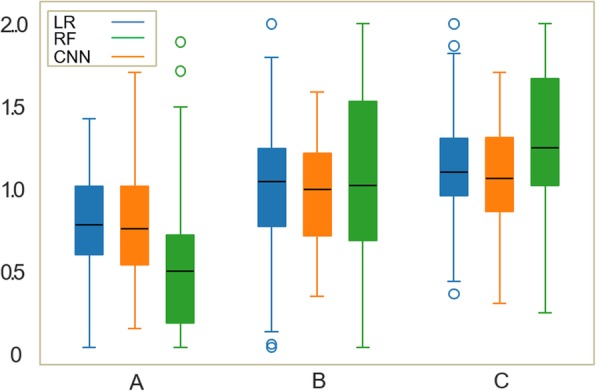
Box-and-whisker plot of the continuous rating scores obtained through linear regression (LR), random forest (RF) and convolution neural network (CNN) for patients of each Child-Pugh class. Thick black horizontal bars, boxes, whiskers, and circles correspond to median, interquartile range, 10th or 90th percentile, and outliers, respectively.

**Fig. 3:**
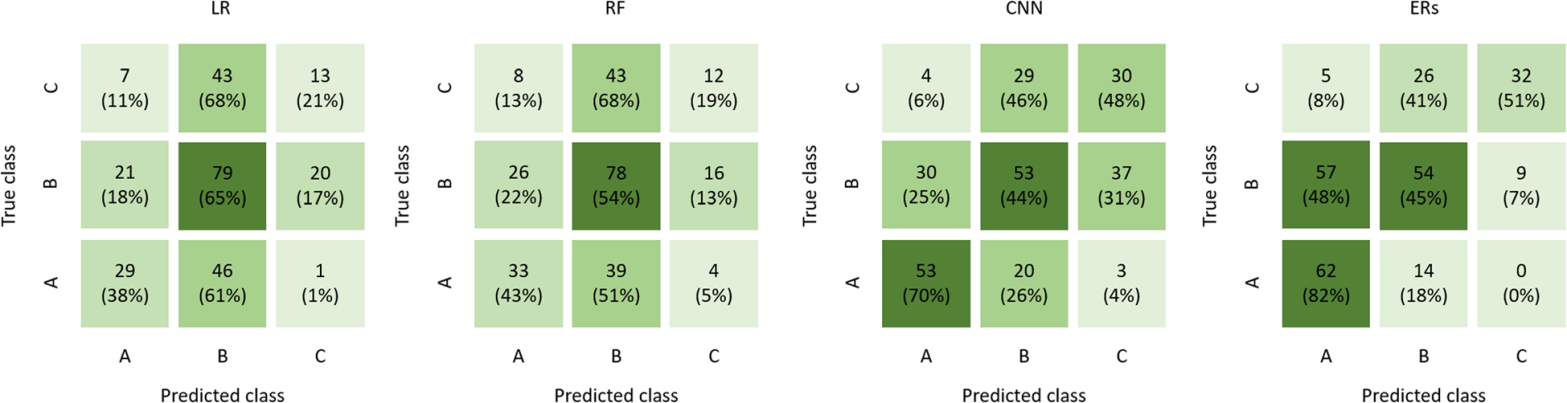
Confusion matrix for the prediction of the Child-Pugh class. From left to right: linear regression (LR), random forest (RF), convolution neural network (CNN), and experienced radiologists (ERs). Values in brackets are relative to the numbers of patient in the ground truth Child-Pugh class.

The results of the RS obtained by the machine learning algorithms for Child-Pugh classes A, B, and C are given in Fig. 2. Spearman correlation coefficient was significant for all algorithms, albeit strongest for CNN. The radiologists’ rating exhibited the strongest correlation (ρ_LR_ = 0.35, ρ_RF_ = 0.32, ρ_CNN_ = 0.51, ρ_ERs_ = 0.60; all *p* < 0.001). The predictivity is illustrated by means of a confusion matrix in Fig. 3. The interobserver agreement between radiologists was *κ* = 67%.

**Fig. 4:**
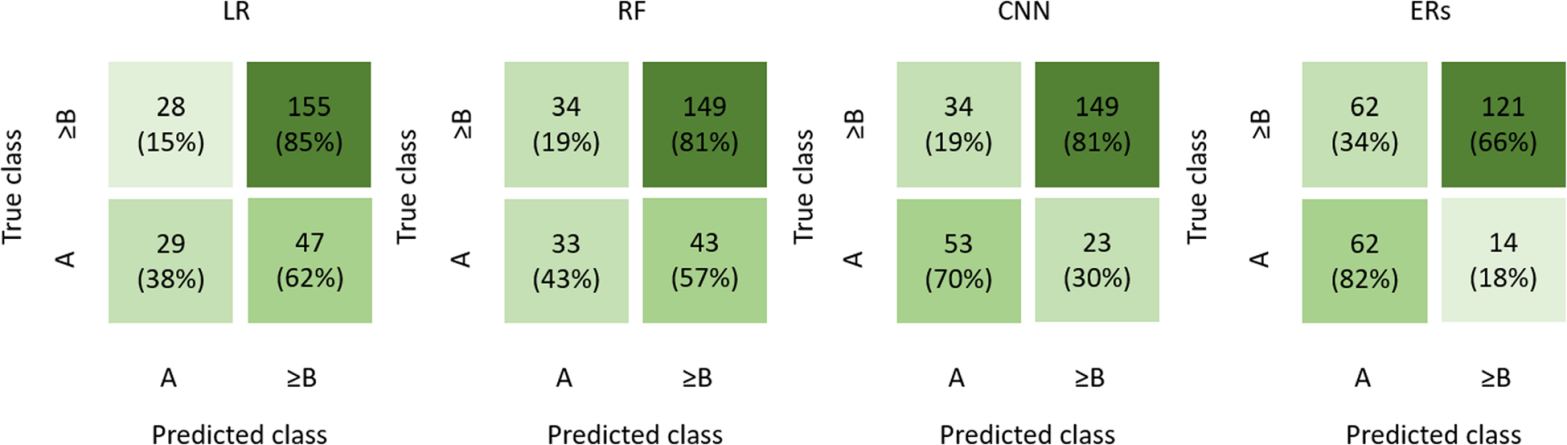
Confusion matrix for the prediction of low disease severity (Child-Pugh class A) and advanced disease severity (Child-Pugh class ≥ B). Linear regression (LR), random forest (RF), convolution neural network (CNN), and experienced radiologists (ERs). Values in brackets are relative to the numbers of patient in the ground truth Child-Pugh class.

The accuracy of the CNN and ERs was significantly better as compared to the no- information-rate (ACC_LR_ = 47%, *p* = 0.477; ACC_RF_ = 47%, *p* = 0.384; ACC_CNN_ = 53%, *p* = 0.034; ACC_ERs_ = 57%; *p* < 0.001; no-information-rate = 46%) (Fig. 3). If binary classification (Child-Pugh class A *versus* Child-Pugh classes B and C) was performed, only the CNN revealed better results against the no-information-rate (Fig. 4):

- LR: accuracy 71%, sensitivity 85%, specificity 38%, *p* = 0.483;
- RF: accuracy 70%, sensitivity 81%, specificity 43%, *p* = 0.579;
- CNN: accuracy 78%, sensitivity 81%, specificity 70%, *p* < 0.001;
- ERs: accuracy 71%, sensitivity 82%, specificity 66%, *p* = 0.531).

**Fig. 5:**
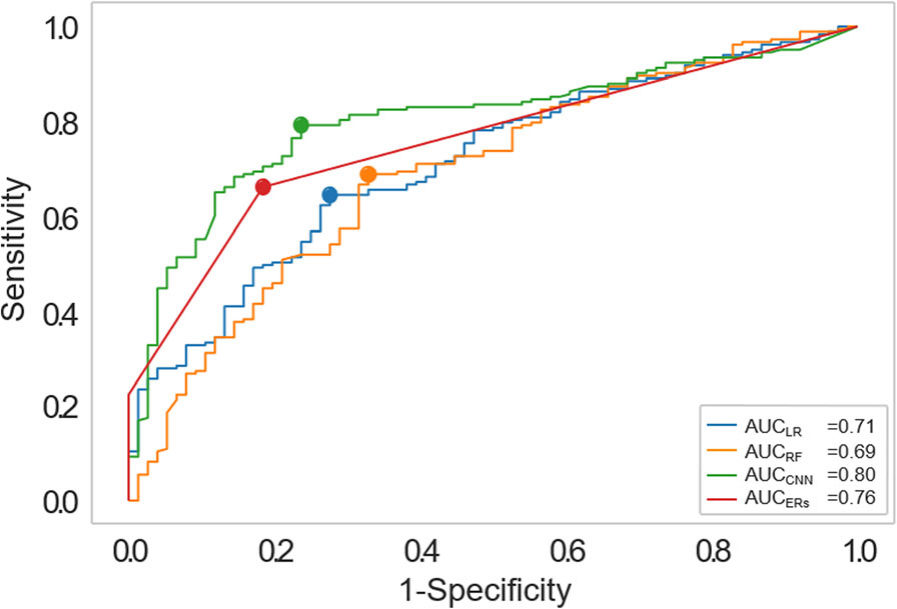
Receiver operating characteristics curves demonstrating the accuracy of the four predictive models in the binary prediction for 256 patients. *LR* Linear regression (blue line*), RF* Random forest (orange line), *CNN* Convolution neuronal network (green line), ERs Experienced radiologists (red line).

The ROC-AUC was highest for CNN (0.80), followed by the ERs prediction (0.76), with AUC interpolated as shown in Fig. 5. However, this difference was not significant (*p* = 0.144). Both the LR classifier (AUC 0.71) and the RF classifier (AUC 0.69) performed significantly worse than either the ERs (*p* = 0.042 as compared to LR and *p* = 0.023 as compared to RF) or the deep learning, *i.e*., CNN, classifier (*p* = 0.041 as compared to LR and *p* = 0.014 as compared to RF) (Table 4 and Fig. 5).

**Table 4.** Accuracy scores for all predictive models

Predictive model	LR	RF	CNN	ERs
Prediction of the Child-Pugh class				
Spearman ρ	0.35	0.32	0.51	0.60
Accuracy (%)	47	47	53	57
Classification Child-Pugh class A *versus* ≥ B				
Accuracy (%)	71	70	78	71
Sensitivity (%)	85	81	81	82
Specificity (%)	38	43	70	66
AUC	0.71	0.67	0.80	0.76

*AUC* Area under the curve at receiver operating characteristic analysis, *LR* Linear regression, *RF* Random forest, *CNN* Convolution neuronal network, *ERs* Experienced radiologists

## Discussion

Our most important finding is that CNN can predict Child-Pugh class, as a surrogate for the severity of liver cirrhosis, with a comparable accuracy to that of ERs (ρ 0.51 and accuracy 53% *versus* ρ 0.60 and accuracy 57%, respectively) based on a clinical multiphase CT. Both conventional radiomic analyses trail these performances in all assessed diagnostic scores. Even though CT has been described as a valid tool to assess distinct morphological changes of liver parenchyma [33], the value of multiphase liver CT in staging liver fibrosis has remained restricted due to its limited functional information that is accessible to the eyes of even trained radiologists [34, 35]. To overcome those limitations, recent studies investigated more functional-based imaging modalities, in particular magnetic resonance imaging [8, 36]. Yasaka et al. implemented a CNN model for the staging of liver fibrosis using gadoxetic acid–enhanced hepatobiliary phase imaging [9], resulting in a good noninvasive prediction of the liver fibrosis grade (ρ 0.63 and AUC 0.80, *p < 0*.001).

The fact that conventional machine learning techniques are outperformed by suitable neural networks is also in line with previous research by our group [28]. Furthermore, other magnetic resonance imaging studies reported, that quantitative texture analyses using T2-weighted images [37] and extracellular gadolinium-enhanced images [38] resulted in nearly the same prediction of liver fibrosis with AUCs of 0.81 and 0.80, respectively. Several other noninvasive modalities have been evaluated for the staging of liver fibrosis; in this regard, ultrasound and magnetic-resonance elastography seem to be promising techniques, which are increasingly applied in clinical practice. However, CT remains the more robust imaging technology which is less severely plagued by obesity, ascites, or the presence of metallic implants [39].

Even though eleven imaging features were shown to be significantly correlated to the Child-Pugh class, the predictions by radiomic analysis (LR and RF) were less accurate than those by CNN or ERs. This is in line with recent literature, indicating that CNNs with their ability to inherently learn features and process implicit imaging information are more suitable for the analysis of medical imaging [40–42]. The radiological assessment of Child-Pugh class resulted in a comparable level of accuracy, therefore we found no proof that machine learning approaches can outperform human experts in the assessment of diffuse liver parenchymal changes.

However, ERs rated the patients with access to the full original clinically used image data. Therefore, context information (*e.g*., presence of portal hypertension, general appearance of the patient) was available to the radiologist and yielded additional information that was not accessible to the radiomic approaches which only received the segmented liver as inputs. The CNNs on the other hand had access to the same full image volume as ERs. This certainly contributes to the superiority of the CNNs as compared to the radiomic approaches. It should be mentioned that we did not purposefully restrict the radiomic approaches to only have access to the segmented liver—rather, this is an inherent requirement of these methods as they calculate radiomic features (among those volume and surface) based on given segmentations only.

Our data are derived from a homogeneous, single-institution cohort of patients with and without diffuse liver parenchyma changes and include the corresponding laboratory and clinical parameters for the calculation of Child-Pugh score and imaging characterisation with robust quantitative analysis, which overcomes methodological shortcomings of alternative user-dependent semiquantitative or qualitative analyses. Bringing machine learning algorithms into clinical practice has been difficult due to differences between the way images are acquired at different centres. One limitation is the potential variability of radiomic features when using different protocols [43] and further research is needed to address this issue.

Another aspect is the need for clinical validation, that we hope to partly address with this manuscript. Future studies will also focus on the clinical application of our algorithms in everyday use and we hope to increase the use of image data in the context of systematic or chronical diseases such as liver cirrhosis.

However, this study has limitations. First, class imbalances can potentially have a negative effect on fitting of machine learning models and were present in the epidemiologic data and the Child-Pugh score. Due to the retrospective nature of this study and the inclusion criteria, only patients with a serious abdominal disease had been included. However, class imbalances were mitigated during training by using subsampling techniques that reduce the majority class and synthesise new data points in the minority classes [31].

Second, generalisation of machine learning algorithms to different scanner manufacturers and acquisition protocols remains a challenging problem that is currently under active investigation [43, 44]. Future research in machine learning will have to address the transferability of such algorithms as proposed in our study.

Another limitation is the high number of excluded patients in our cohort. For the majority (n = 451) of patients, albumin was not acquired as it is not a standard of care parameter, determining a selection bias: only patients whose blood samples were analysed for albumin levels were incorporated. Furthermore, the reports of the cognitive status were derived from standardised daily bedside records. Although deviations in the mental status have to confirmed or revealed by a specialised neurologist, it is possible that especially mild stages of encephalopathy could have been missed.

In addition, we should consider that liver cirrhosis is a heterogeneous disease that consequently leads to a wide spectrum of patients with differing underlying causes for those changes (*e.g*., virus infection, metabolism disorders, drug exposure, side effects of medication, etc.). Therefore, the Child-Pugh classification might not be the perfect measure to assess the severity of liver fibrosis. Whether this clinical categorisation of disease severity leads to weak predictions for all models remains to be investigated. Also, the use of three-dimensional CNN deserves future investigations requiring a greater number of patients, being the number of parameters higher than that considered for two-dimensional CNN.

Finally, we note that invasive liver biopsy is still the standard of care for diagnosis and grading liver cirrhosis. However, image data can yield additional information that is accessible noninvasively, thus easier to acquire and can be considered to supplement clinically established laboratory parameters. Thus, machine learning algorithms—in particular CNN—may provide additional quantitative and objective information to assess functional liver status based on clinical CT images.

## Data Availability

The datasets used and analysed during the current study are available from the corresponding author on reasonable request.

## Abbreviations

AUC: Area under the curve
CNN: Convolutional neural network
CT: Computed tomography
ERs: Experienced radiologists
LR: Linear regression
MELD: Model of end-stage liver disease
RF: Random forest
ROC: Receiver operating characteristics
RS: Rating score
